# Early C-reactive protein reduction predicts survival in real-world extensive-stage small cell lung cancer treated with first-line adebrelimab-based immunotherapy

**DOI:** 10.3389/fonc.2025.1709336

**Published:** 2025-11-05

**Authors:** Jian Wang, Xueli Zhang, Qijia Gao, Jianxin Chen

**Affiliations:** ^1^ Department of International Ward, The Quzhou Affiliated Hospital of Wenzhou Medical University, Quzhou People′s Hospital, Quzhou, Zhejiang, China; ^2^ Department of Gastroenterology, Jiaxing Second Hospital, Jiaxing, Zhejiang, China; ^3^ Department of Pediatrics, Department of Medical Oncology, The Quzhou Affiliated Hospital of Wenzhou Medical University, Quzhou People′s Hospital, Quzhou, Zhejiang, China; ^4^ Changsha Medical University, Changsha, Hunan, China

**Keywords:** extensive-stage small cell lung cancer, C-reactive protein, real-world evidence, adebrelimab, prognostic biomarker

## Abstract

**Background:**

Extensive-stage small cell lung cancer (ES-SCLC) remains an aggressive malignancy with limited biomarkers for predicting outcomes in real-world settings. While baseline systemic inflammation correlates with prognosis, the role of longitudinal inflammation dynamics during PD-L1 inhibitor-based therapy is unexplored. This study investigated whether early changes in systemic inflammation markers, particularly C-reactive protein (CRP), predict clinical efficacy in ES-SCLC patients receiving first-line adebrelimab plus chemotherapy.

**Methods:**

In this retrospective, single-center study, 35 ES-SCLC patients (median age: 72 years) treated with adebrelimab plus platinum-etoposide or platinum-irinotecan chemotherapy were analyzed. Ten systemic inflammation markers (NLR, PLR, LMR, PAR, SII, NPR, CAR, CLR, CRP, LDH) were assessed at baseline and after 2 months of therapy. Inflammatory trends were quantified as the ratio of 2-month to baseline values. Associations between inflammation dynamics and survival (OS from 2 months, OS2) or radiologic response (RECIST 1.1) were evaluated using Kaplan-Meier analysis, Cox regression, and Spearman’s correlation.

**Results:**

The cohort showed robust real-world efficacy (median OS: 15.0 months; ORR: 62.8%). Among ten inflammation markers analyzed, only CRP dynamics were significantly associated with OS in univariate analysis. Patients achieving CRP reduction (trend ratio <1) at 2 months had significantly longer median OS (16.2 months) versus those without reduction (8.1 months; HR = 3.492, 95% CI:1.239–9.847, P = 0.011). No other inflammatory trend correlated with OS. Inflammation dynamics (including CRP) showed no association with best overall response or tumor regression (P>0.05 for all markers).

**Conclusion:**

Early reduction in CRP levels during adebrelimab-based chemoimmunotherapy is an potentially predictor of improved survival in ES-SCLC, despite dissociation from initial radiologic response. This suggests that CRP kinetics could serve as a practical, real-world biomarker for prognostication and early efficacy assessment in ES-SCLC. Prospective validation in larger cohorts is essential to confirm these findings.

## Introduction

Extensive-stage small cell lung cancer (ES-SCLC) remains a therapeutic challenge characterized by aggressive biology and dismal prognosis (1, 2). The integration of programmed death-ligand 1 (PD-L1) inhibitors with platinum-etoposide chemotherapy has redefined first-line treatment, demonstrating significant survival improvements in phase III trials such as IMpower133 (atezolizumab), CASPIAN (durvalumab), and CAPSTONE-1 (adebrelimab) (3–5). Adebrelimab (SHR-1316), a novel humanized anti-PD-L1 monoclonal antibody, emerged as a standard-of-care option in China following the CAPSTONE-1 trial, which reported a median overall survival (OS) of 15.3 months with adebrelimab plus chemotherapy versus 12.8 months with chemotherapy alone (5).

While pivotal trials establish efficacy under controlled conditions, real-world evidence (RWE) is indispensable for validating outcomes in unselected populations excluded from randomized studies—particularly older patients, those with elevated comorbidity burdens, or suboptimal performance status (6, 7). Our initial real-world study (currently under review; data on file) evaluated first-line adebrelimab-based therapy in 35 ES-SCLC patients. This analysis confirmed robust real-world efficacy (median OS: 15.0 months; median progression-free survival [PFS]: 7.1 months) despite a cohort median age of 72 years, aligning with CAPSTONE-1 outcomes (5). Critically, we identified baseline Eastern Cooperative Oncology Group Performance Status (ECOG PS) ≥2, metastatic burden (≥2 organs), and elevated C-reactive protein (CRP ≥5 mg/L) as potential prognostic factors for survival. The association between baseline CRP elevation and inferior OS (HR = 3.337; p=0.044) underscores systemic inflammation’s role in ES-SCLC progression-a finding consistent with mechanisms linking interleukin-6-driven inflammation to immunosuppression and tumor aggressiveness (8–11).

Nevertheless, static biomarker assessments at diagnosis provide an incomplete picture of the dynamic host-tumor-immune interplay during immunotherapy. ​Mounting evidence suggests that longitudinal changes in systemic inflammation may more accurately predict therapeutic outcomes than baseline values alone​ (12–14). In non-small cell lung cancer (NSCLC), early CRP reduction after immune checkpoint inhibitor (ICI) initiation correlates significantly with improved OS and PFS, suggesting utility as a pharmacodynamic biomarker (12, 13). Whether such ​dynamic inflammation monitoring holds predictive value in ES-SCLC-where tumor microenvironments exhibit distinct neuroendocrine features and heightened immunosuppression-remains unexplored​ (15).

To address this gap, we leveraged our previously characterized real-world cohort to conduct a focused biomarker substudy. ​This analysis specifically investigates (1): the longitudinal dynamics of ten systemic inflammation markers (including hematologic ratios, CRP-derived indices, and lactate dehydrogenase) during adebrelimab-based therapy; and (2) their association with radiologic response and survival outcomes.​​ We hypothesized that early modulation of systemic inflammation-particularly CRP dynamics-would correlate with clinical efficacy, providing a readily accessible tool for real-world prognostication.

## Methods

### Study design and patient population

This retrospective, single-center study analyzed 35 extensive-stage small cell lung cancer (ES-SCLC) patients treated with first-line adebrelimab plus chemotherapy (etoposide/carboplatin, etoposide/cisplatin, or irinotecan/cisplatin) at Quzhou People’s Hospital (September 2021 to March 2025). ​The primary efficacy and safety outcomes of this cohort were previously reported​ (Chen et al., under review). The present analysis focused exclusively on systemic inflammation dynamics and their prognostic impact. Key inclusion/exclusion criteria mirrored the initial study. Ethical approval was obtained from the Quzhou People’s Hospital Ethics Committee, adhering to the Declaration of Helsinki.

### Systemic inflammation assessment

Ten systemic inflammation markers were longitudinally evaluated at two critical timepoints: baseline (prior to treatment initiation) and after 2 months of therapy. The 2-month timepoint was selected based on clinical practice, where the first radiological assessment typically occurs after 2–3 cycles of therapy. These markers encompassed as follows. ​Hematologic ratios included Neutrophil-to-lymphocyte ratio (NLR), platelet-to-lymphocyte ratio (PLR), lymphocyte-to-monocyte ratio (LMR), platelet-to-albumin ratio (PAR), and neutrophil-to-platelet ratio (NPR). ​CRP-derived ratios included C-reactive protein (CRP)-to-albumin ratio (CAR) and CRP-to-lymphocyte ratio (CLR). ​Composite indices included Systemic Immune-Inflammation Index (SII), calculated as platelets × neutrophils/lymphocytes. ​Direct biomarkers included Serum CRP (mg/L) and lactate dehydrogenase (LDH; U/L). Cutoff values were based on established clinical thresholds (8, 10).

### Key operational definitions

Temporal dynamics: For each marker, baseline values were denoted as “bMarker” (e.g., bNLR, bCRP), while 2-month values were labeled “Marker2” (e.g., NLR2, CRP2). The inflammatory trend was quantified as the ratio of Marker2 to its corresponding baseline value (i.e., trend = Marker2/bMarker).

​Inflammation improvement: Improvement status was dichotomized based on directionally consistent physiological expectations. For NLR, PLR, PAR, SII, NPR, CAR, CLR, CRP, and LDH, improvement was defined as a trend ratio < 1, indicating a decrease from baseline. For LMR exclusively, improvement was defined as a trend ratio > 1, reflecting an increase from baseline.

​Baseline stratification: Patients were categorized into high versus low inflammation subgroups using predefined, clinically established cutoff values for each marker (summarized in **Table 1**).

**Table 1:** Cutoff values used to define high inflammation levels for each biomarker at baseline.

Inflammation levels	Cut-off value
NLR	≥3
PLR	≥200
LMR	<2
PAR	≥7
SII	≥800
NPR	≥0.02
CAR	≥0.1
CLR	≥10
CRP	≥5
LDH	≥250

NLR, Neutrophil-to-Lymphocyte Ratio;PLR, Platelet-to-Lymphocyte Ratio; LMR, Lymphocyte-to-Monocyte Ratio; PAR, Platelet-to-Albumin Ratio; SII, Systemic Immune-Inflammation Index; NPR, Neutrophil-to-Platelet Ratio; CAR, C-reactive protein-to-Albumin Ratio; CLR, C-reactive protein-to-Lymphocyte Ratio; CRP, C-reactive Protein; LDH, Lactate Dehydrogenase.

### Statistical analysis

The primary endpoints for this analysis were defined as follows. ​Overall survival from 2 months post-treatment initiation (OS2)​​ was evaluated to assess survival outcomes beyond the initial treatment phase. Additionally, ​inflammation improvement rates​ were calculated for each marker, representing the percentage of patients demonstrating a reduction (or increase, for LMR) in inflammatory markers from baseline to 2 months. To compare OS2 based on inflammation trends, ​Kaplan-Meier survival curves​ were generated, and between-group differences were assessed using the ​log-rank test​. This non-parametric method evaluates whether the survival distributions of groups (e.g., patients with *vs*. without CRP improvement) are statistically distinct, with significance determined by a p-value ≤ 0.05. Due to the limited sample size, only ​univariate Cox regression analyses​ were performed for each inflammatory trend variable to avoid overfitting. Cox proportional hazards regression was employed to assess univariate associations between each inflammatory trend variable and overall survival, with results presented as hazard ratios (HR) and 95% confidence intervals (CI). The model incorporated clinically relevant covariates (age, ECOG performance status, metastatic burden) along with all inflammatory trend variables (e.g., NLR, PLR, CRP trends). Hazard ratios (HR) with 95% confidence intervals (CI) were reported to quantify the magnitude and direction of associations. Associations between categorical variables-specifically, inflammation trends (dichotomized as improved *vs*. not improved) and best overall response (complete/partial response *vs*. stable/progressive disease per RECIST 1.1 (16, 17))-were evaluated using ​Chi-square tests​ or ​Fisher’s exact tests​. The choice between tests depended on expected frequencies: Fisher’s exact test was used for small sample sizes or sparse data (e.g., >20% of cells with expected counts <5), while Chi-square was applied for larger tables. To quantify the relationship between continuous variables, ​Spearman’s rank correlation​ analysis was performed. This non-parametric method assessed monotonic associations between the percentage change in inflammatory markers and the degree of tumor regression (expressed as percentage change from baseline). Spearman’s ρ (rho) values ranging from -1 to 1 were interpreted, with positive values indicating parallel changes and negative values indicating inverse relationships. All statistical analyses were conducted using ​SPSS software (version 23.0)​. A two-sided ​significance threshold of p ≤ 0.05​ was applied for all tests. Given the small sample size (n=35) and retrospective design, the findings should be interpreted as exploratory and hypothesis-generating, requiring validation in larger cohorts.

## Results

### Patient characteristics and treatment outcomes

Thirty-five patients with advanced SCLC were included in this study. The median age was 72 years, and most were male (88.6%) with a smoking history (68.6%). The majority had good performance status (PS 0–1, 85.7%). Nearly half of the patients (48.6%) presented with fewer than two metastatic sites, while 51.4% had two or more, including 31.4% with brain metastases. Patients received adebrelimab in combination with EC (28.6%), EP (51.4%), or IP (20%), with a median of 4 cycles administered.

**Figure 1:**
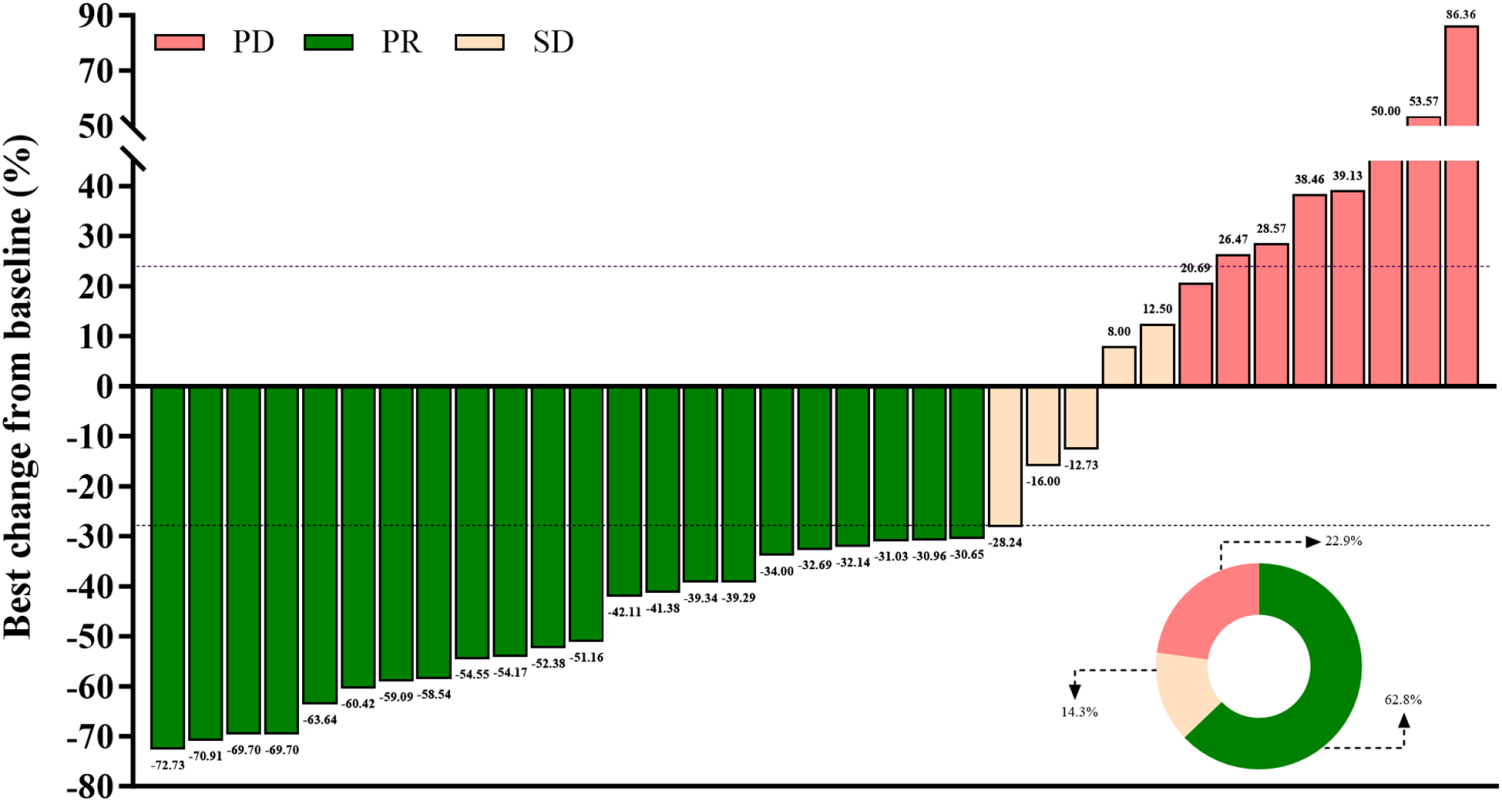
Best tumor size changes: PR (green), SD (beige), PD (pink).

As shown in **Figure 1**, the waterfall plot displays tumor shrinkage. For treatment response, 22 patients (62.8%) achieved partial response (PR), 5 (14.3%) achieved stable disease (SD), and 8 (22.9%) experienced progressive disease, resulting in an ORR of 62.8% and a DCR of 77.1%. The degree of tumor shrinkage among PR patients ranged from 72.73% to 30.65%, as illustrated in the waterfall plot (**Figure 1**). Median progression-free survival (PFS) was 7.1 months (95% CI: 5.47–8.53), and median overall survival (OS) was 15.0 months (95% CI: 10.47–19.53; **Supplementary Figure S1**).

### Inflammation improvement after adebrelimab-based treatment

**Figure 2:**
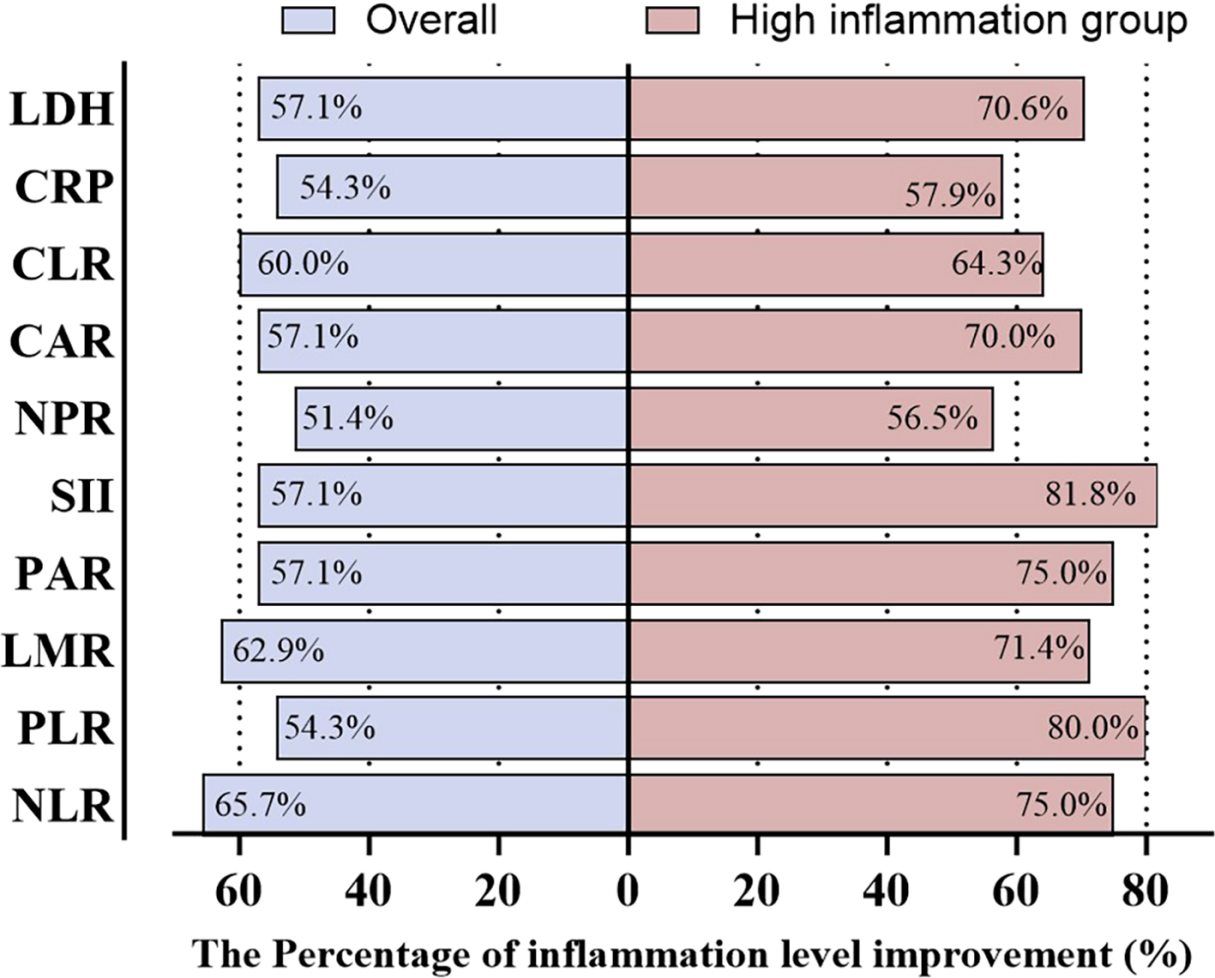
Improvement rates in inflammation after 2 months of treatment.

After 2 months of Adebrelimab-based treatment, the overall cohort showed inflammation improvement rates ranging from 51.4% to 65.7%, with the highest improvement observed in NLR (65.7%). In the high-inflammation subgroup, improvement rates ranged from 57.9% to 81.8%, with SII showing the greatest improvement (81.8%). **Figure 2** showed that the high-inflammation group demonstrated consistently higher improvement rates across all markers compared with the overall population.

### Prognostic factors and subgroup analysis for OS

**Figure 3:**
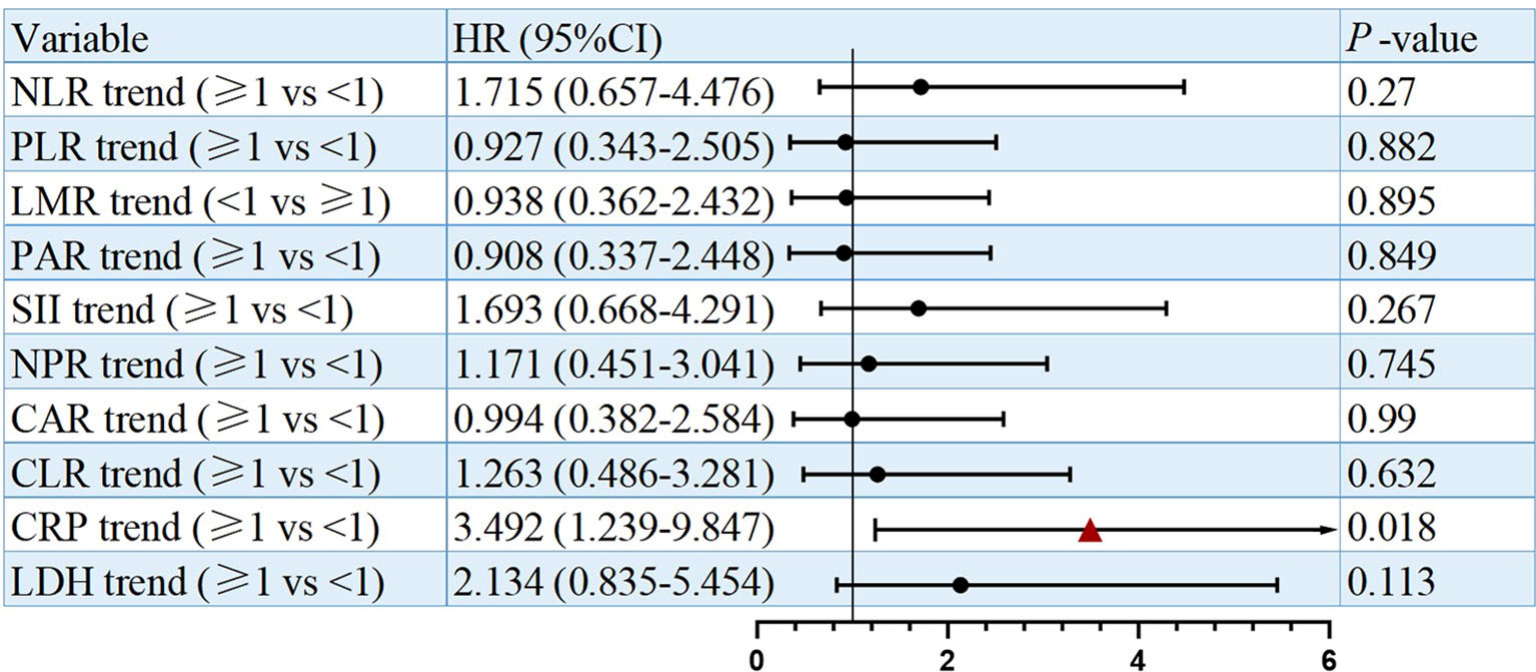
Univariate analysis of prognostic factors for OS.

**Figure 4:**
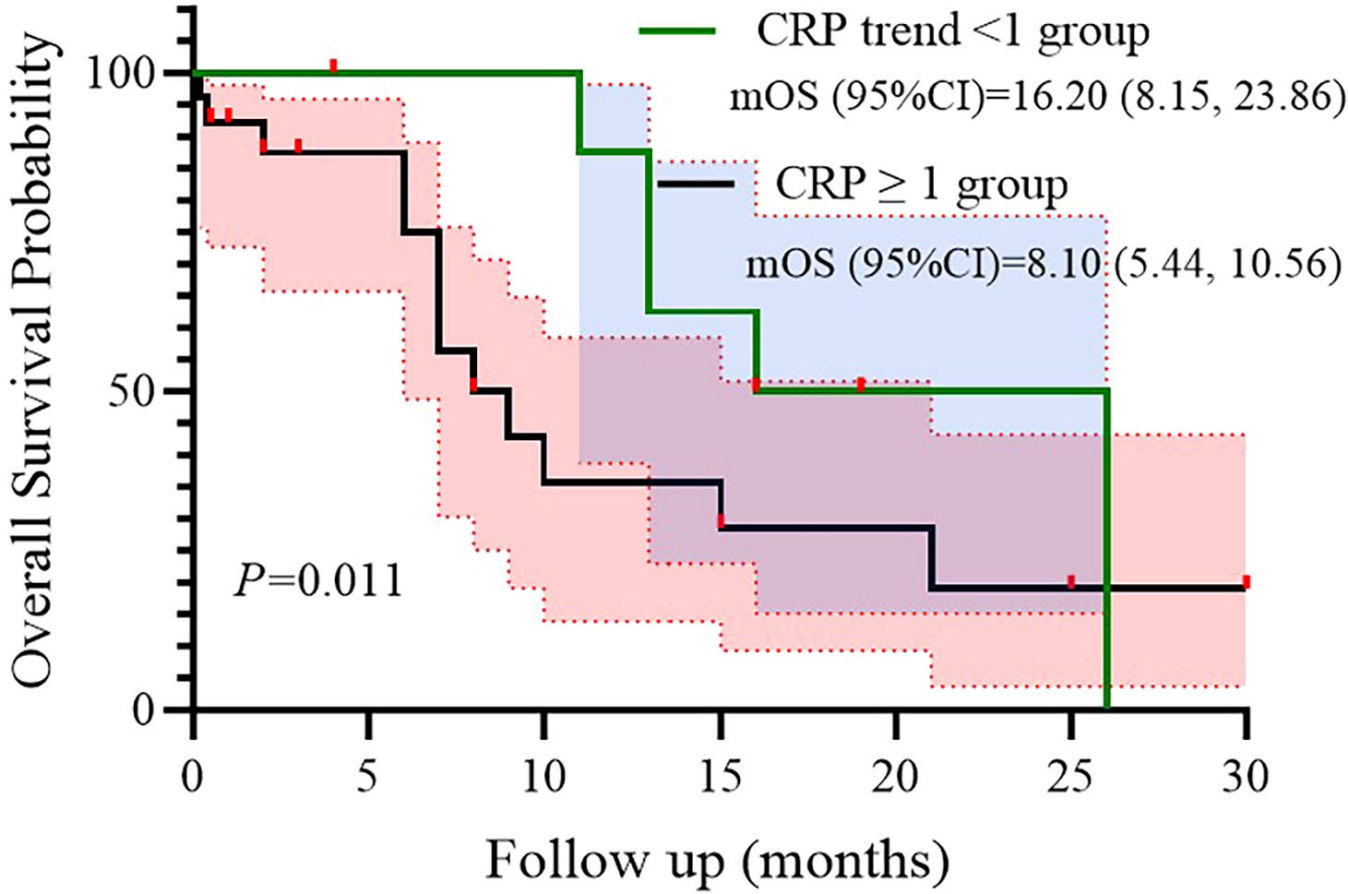
Subgroup analysis of CRP trend as prognostic factor for OS.

In survival analysis, CRP dynamics were significantly associated with OS in univariate analysis, with a hazard ratio of 3.492 (95% CI: 1.239–9.847, P = 0.018; **Figure 3**). Kaplan–Meier analysis further suggested that patients with CRP trend <1 had a significantly longer median OS (16.2 months, 95% CI: 8.15–23.86) compared to those with CRP trend ≥1 (8.1 months, 95% CI: 5.44–10.56, P = 0.011; **Figure 4**). Besides, other inflammatory biomarker trends were not significantly associated with OS.

### Response by inflammatory-marker trends

**Figure 5:**
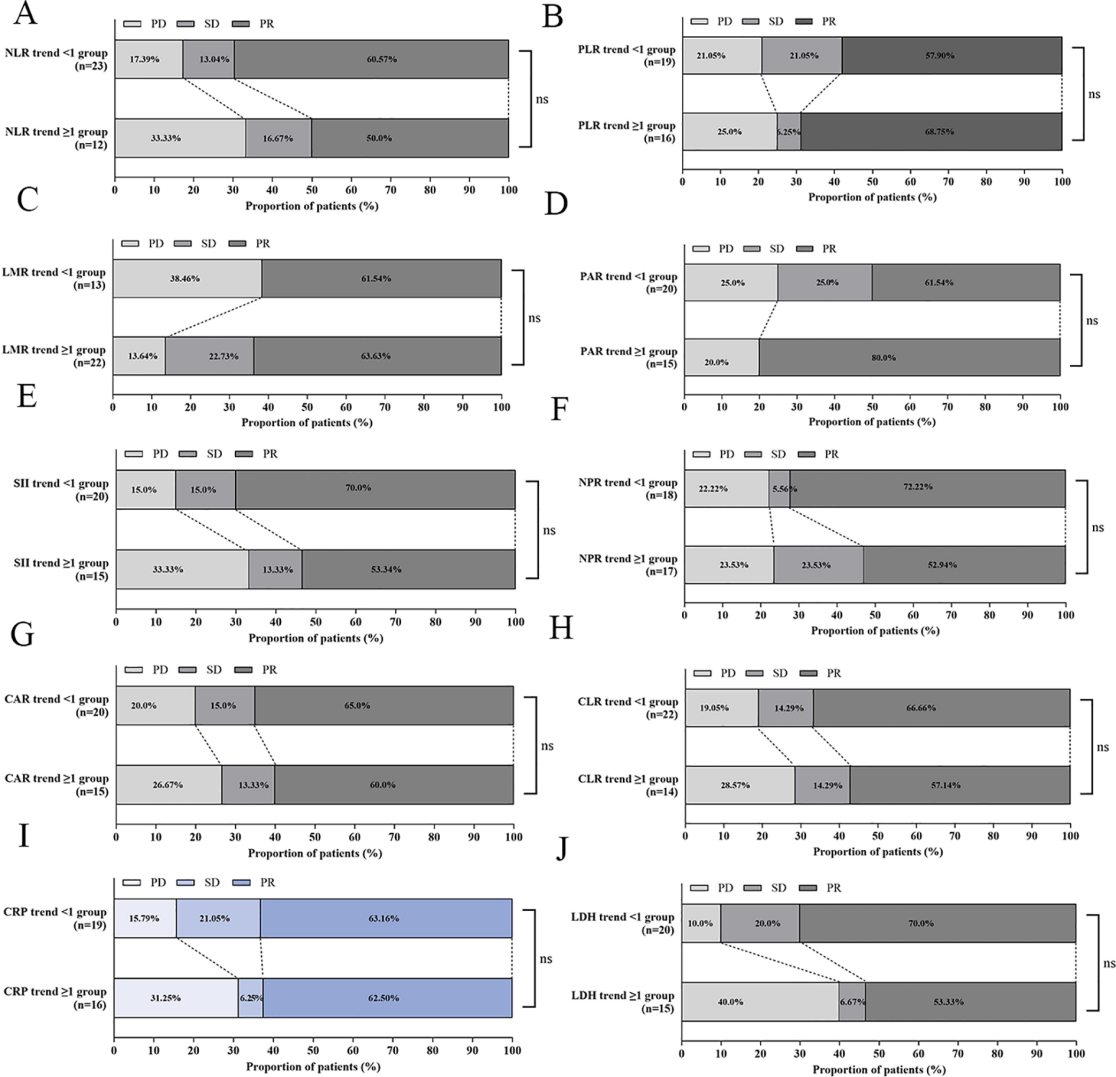
Distribution of best overall response by inflammatory-marker trends. Stacked bar charts for **(A)** LR, **(B)** PLR, **(C)** LMR, **(D)** PAR, **(E)** SII, **(F)** NPR, **(G)** CAR, **(H)** CLR, **(I)** CRP, and **(J)** LDH.

For all the ten indices–NLR, PLR, LMR, PAR, SII, NPR, CAR, CLR, CRP, and LDH–the distributions of radiologic response (PR/SD/PD) were similar between the two trend groups; none of the comparisons was statistically significant (*all p* > 0.05, **Figure 5**).

### Correlation between inflammatory-marker changes and tumor regression

**Figure 6:**
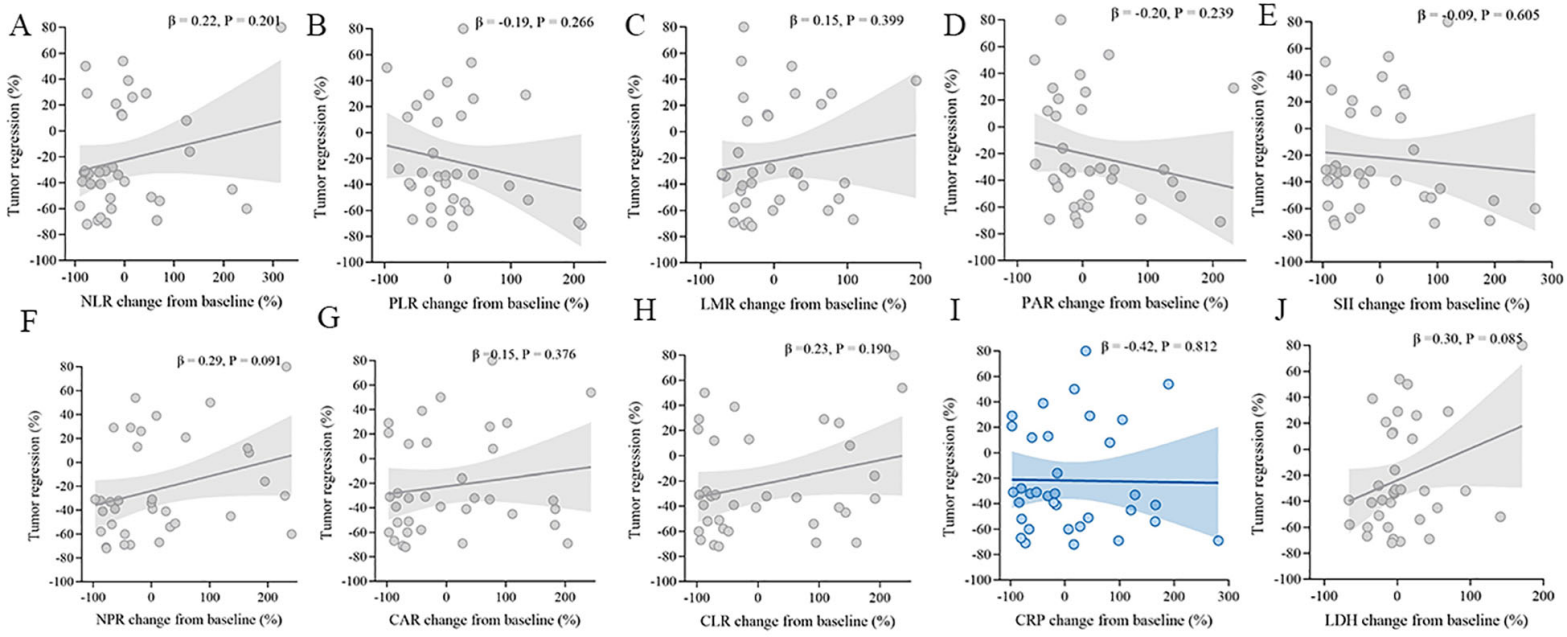
Correlations between post-treatment changes in inflammatory markers and tumor regression. Scatter plots with linear fit and 95% CI for **(A)** NLR, **(B)** PLR, **(C)** LMR, **(D)** PAR, **(E)** SII, **(F)** NPR, **(G)** CAR, **(H)** CLR, **(I)** CRP, and **(J)** LDH.

Across all ten indices (NLR, PLR, LMR, PAR, SII, NPR, CAR, CLR, CRP, and LDH), no significant correlations were observed between the percentage change in the marker and tumor regression (*all p* > 0.05, **Figure 6**).

## Discussion

This real-world biomarker study provides novel insights into the prognostic utility of longitudinal systemic inflammation dynamics during first-line adebrelimab-based immunotherapy for ES-SCLC. Our core finding that a decline in CRP levels after 2 months of treatment might predicts significantly improved survival, highlights the dynamic interplay between host inflammation and therapeutic efficacy, offering a clinically accessible prognostic tool. This is particularly relevant in ES-SCLC, where aggressive biology and limited biomarkers challenge personalized management. However, due to the small sample size, multivariable modeling including all variables was avoided to prevent overfitting. The univariate analyses should be interpreted as exploratory.

We confirmed robust real-world efficacy of adebrelimab-chemotherapy in an older, unselected cohort (median OS: 15.0 months), aligning with CAPSTONE-1 trial data despite higher median age (72 *vs*. 62 years) (5). More critically, we extended prior observations linking baseline systemic inflammation (e.g., CRP ≥5 mg/L) to poor prognosis (8–11) by demonstrating that longitudinal CRP dynamics during treatment serve as a potential survival predictor. Patients achieving CRP reduction (trend ratio <1) at 2 months had a median OS of 16.2 months versus 8.1 months for those without reduction (HR = 3.492, p=0.011). This underscores that early modulation of host inflammation, rather than its static baseline state, is a key determinant of survival. The accessibility and low cost of CRP measurement position it as a practical real-world biomarker for risk stratification and early efficacy assessment.

​While baseline CRP elevation predicted inferior OS (HR = 3.337, p=0.044) in our prior analysis, In univariate analysis, CRP dynamics emerged as a prognostic factor (HR = 3.492, p=0.018), even after adjusting for ECOG PS, metastatic burden, and age. This exploratory finding suggests CRP dynamics may have prognostic value in ES-SCLC. This suggests that on-treatment inflammation resolution reflects effective restoration of antitumor immunity (18, 19). Mechanistically, IL-6-driven CRP production is linked to immunosuppressive myeloid cell expansion and PD-L1 upregulation in SCLC (8, 11, 15). Adebrelimab’s PD-L1 blockade may reverse this suppression more effectively in responders, manifesting as CRP decline. Besides, the lack of significant prognostic associations for the other nine inflammation markers (NLR, PLR, LMR, PAR, SII, NPR, CAR, CLR, LDH) or their trends is noteworthy. This contrasts with NSCLC studies where NLR or CAR dynamics correlate with ICI outcomes (12–14). SCLC exhibits a unique tumor microenvironment characterized by neuroendocrine features and elevated IL-6 signaling, which drives CRP production and immunosuppression. This may explain CRP’s superior prognostic value over other markers in SCLC. Possible explanations include (1): ​SCLC-Specific Microenvironment:​​ SCLC exhibits distinct neuroendocrine features, higher tumor mutational burden, and denser immunosuppressive stroma (15, 20). Furthermore, while SCLC shares a high tumor burden with malignancies such as malignant melanoma, the dynamics of inflammatory markers may differ significantly due to distinct tumor microenvironmental characteristics. For instance, melanoma often exhibits a more immune-inflamed phenotype with higher T-cell infiltration, whereas SCLC is characterized by a neuroendocrine-driven and immunosuppressive stroma that may alter systemic inflammation responses. This distinction could explain why certain biomarkers (e.g., CRP or NLR) show varying prognostic values across cancer types. Comparative studies across neoplasms are needed to validate these observations, as suggested in recent literature (21, 22). CRP, as an acute-phase reactant directly regulated by IL-6, may more sensitively reflect systemic immunosuppression reversal upon PD-L1 blockade compared to hematologic ratios influenced by multiple confounders (e.g., bone marrow reserve, occult infections in elderly patients) (2). ​Threshold Effects:​​ While most markers showed improvement rates exceeding 50% in high-inflammation subgroups (e.g., SII: 81.8%), these changes lacked survival correlation. This implies that reduction in these ratios, though common, may not consistently reflect biologically meaningful immunomodulation in SCLC.

​In addition, the absence of correlation between inflammation marker dynamics (including CRP trend) and best overall response (**Figure 5**) or tumor regression percentage (**Figure 6**) aligns with emerging understanding of immunotherapy. Clinical benefit from ICIs can occur without immediate tumor shrinkage (e.g., pseudoprogression, immune-related response patterns) (23). CRP decline may thus capture early biological effects on the host environment preceding or independent of measurable tumor burden changes, acting as a pharmacodynamic biomarker rather than a direct predictor of RECIST response. This dissociation reinforces the unique value of dynamic inflammation assessment beyond conventional radiology.

Our findings resonate with, yet crucially extend, prior work as follows (1): ​NSCLC Evidence:​​ Publications demonstrated that early CRP reduction (within 6–8 weeks) predicted superior OS/PFS in NSCLC patients receiving ICIs (13, 14). We establish a parallel phenomenon in ES-SCLC, suggesting that inflammation kinetics may represent a potentially valuable ICI response biomarker across different cancer types, though further validation is needed (2). ​SCLC Inflammation Prognosis:​​ Established researches reported baseline CLR as prognostic in SCLC receiving chemoimmunotherapy (12). We validate the importance of inflammation in SCLC prognosis but demonstrate the dynamic change in CRP (not CLR or baseline values) holds superior predictive power (3). ​Mechanistic Plausibility:​​ The link between IL-6/CRP axis and SCLC aggressiveness/immunosuppression is well-supported (10, 11). Our results provide clinical translation: effective PD-L1 blockade mitigates this inflammation, and measurable CRP decline signifies this therapeutic effect, correlating with survival. This contrasts with former reports (10, 11), where CRP elevation predicted worse outcomes with targeted therapies (erlotinib/pemetrexed), suggesting inflammation’s prognostic role transcends specific therapies but its dynamic modulation is particularly relevant for immunotherapy (24, 25) (4). ​RWE Relevance:​​ Our findings in an elderly, real-world cohort (median age 72 years) complement pivotal trials like CAPSTONE-1 (5), addressing the critical evidence gap for populations underrepresented in RCTs. The consistency of OS benefit (15.0 *vs*. 15.3 months) and the identification of a practical dynamic biomarker enhance the generalizability of adebrelimab efficacy.

This study has inherent limitations of a retrospective, single-center design with a modest sample size (n=35). The small cohort limits the power for extensive multivariable modeling and subgroup analyses (e.g., impact of specific chemotherapy backbones). Blood sampling was restricted to baseline and 2 months; more frequent assessments might reveal finer kinetic patterns or earlier predictive windows. The focus on peripheral blood biomarkers does not capture localized tumor immune microenvironment changes. Validation in larger, multi-center prospective cohorts is essential. Although predefined clinical cutoffs were used, optimization of thresholds for CRP dynamics (trend ratio) in larger datasets could enhance predictive accuracy. In addition, hyperprogression was not assessed due to the cohort size and undefined criteria in SCLC; this warrants investigation in future studies. Furthermore, the potential impact of steroids on inflammation markers in patients with brain metastases was not evaluated and should be considered in future studies. Finally, causality cannot be inferred; CRP reduction may be a surrogate for other biological processes driving better outcomes.

This suggests that CRP kinetics could serve as a practical, real-world biomarker for prognostication and early efficacy assessment in ES-SCLC. This finding underscores the potential role of modulating systemic inflammation-specifically the IL-6/CRP axis-in achieving clinical benefit from PD-L1 inhibition in SCLC. While numerous hematologic and composite inflammation indices were longitudinally assessed, CRP dynamics demonstrated potential prognostic value, dissociated from immediate radiologic tumor response but powerfully linked to long-term survival. The simplicity and wide availability of CRP measurement position it as a highly practical tool for real-world prognostication and early efficacy assessment in ES-SCLC management. Prospective, multicenter studies with larger cohorts are essential to validate these hypothesis-generating findings and translate CRP kinetics into clinically actionable biomarkers.

## Data Availability

The original contributions presented in the study are included in the article/**Supplementary Material**. Further inquiries can be directed to the corresponding author.

## References

[1] Li M Chen YZ Chen DZ Wang Y Zhao HL. First-line treatment of extensive-stage small cell lung cancer with immune checkpoint inhibitors acting on different targets: a systematic review and network meta-analysis. Transl Cancer Res. (2025) 14:3961–72. doi: 10.21037/tcr-2025-430, PMID: 40792152 PMC12335701

[2] Jiang Z Zhao F Li B He J Yang H Ji Y. Multidimensional comparative evaluation of first-line therapies for extensive-stage small cell lung cancer: a systematic review and network meta-analysis of clinical efficacy and safety profiles. BMC Cancer. (2025) 25:1292. doi: 10.1186/s12885-025-14750-4, PMID: 40783512 PMC12335103

[3] Horn L Mansfield AS Szczesna A Havel L Krzakowski M Hochmair MJ. First-line atezolizumab plus chemotherapy in extensive-stage small-cell lung cancer. N Engl J Med. (2018) 379:2220–9. doi: 10.1056/NEJMoa1809064, PMID: 30280641

[4] Paz-Ares L Dvorkin M Chen Y Reinmuth N Hotta K Trukhin D. Durvalumab plus platinum-etoposide versus platinum-etoposide in first-line treatment of extensive-stage small-cell lung cancer (CASPIAN): a randomised, controlled, open-label, phase 3 trial. Lancet. (2019) 394:1929–39. doi: 10.1016/S0140-6736(19)32222-6, PMID: 31590988

[5] Wang J Zhou C Yao W Wang Q Min X Chen G. Adebrelimab or placebo plus carboplatin and etoposide as first-line treatment for extensive-stage small-cell lung cancer (CAPSTONE-1): a multicentre, randomised, double-blind, placebo-controlled, phase 3 trial. Lancet Oncol. (2022) 23:739–47. doi: 10.1016/S1470-2045(22)00224-8, PMID: 35576956

[6] Murthy VH Krumholz HM Gross CP. Participation in cancer clinical trials: race-, sex-, and age-based disparities. JAMA. (2004) 291:2720–6. doi: 10.1001/jama.291.22.2720, PMID: 15187053

[7] Unger JM Vaidya R Hershman DL Minasian LM Fleury ME. Systematic review and meta-analysis of the magnitude of structural, clinical, and physician and patient barriers to cancer clinical trial participation. J Natl Cancer Inst. (2019) 111:245–55. doi: 10.1093/jnci/djy221, PMID: 30856272 PMC6410951

[8] Templeton AJ McNamara MG Seruga B Vera-Badillo FE Aneja P Ocana A. Prognostic role of neutrophil-to-lymphocyte ratio in solid tumors: a systematic review and meta-analysis. J Natl Cancer Inst. (2014) 106:dju124. doi: 10.1093/jnci/dju124, PMID: 24875653

[9] Richlitzki C Wiesweg M Metzenmacher M Guberina N Pottgen C Hautzel H. C-reactive protein as robust laboratory value associated with prognosis in patients with stage III non-small cell lung cancer (NSCLC) treated with definitive radiochemotherapy. Sci Rep. (2024) 14:13765. doi: 10.1038/s41598-024-64302-2, PMID: 38877146 PMC11178931

[10] Fiala O Pesek M Finek J Topolcan O Racek J Minarik M. High serum level of C-reactive protein is associated with worse outcome of patients with advanced-stage NSCLC treated with erlotinib. Tumour Biol. (2015) 36:9215–22. doi: 10.1007/s13277-015-3660-3, PMID: 26088452

[11] Fiala O Hosek P Pesek M Finek J Racek J Buchler T. Prognostic role of serum C-reactive protein in patients with advanced-stage NSCLC treated with pemetrexed. Neoplasma. (2017) 64:605–10., PMID: 28485168 10.4149/neo_2017_416

[12] Sheng S Wu Z Zheng H Zhang H Zhang Q Liu Z. Prognostic value of C-reactive protein-to-lymphocyte ratio in combined immunotherapy and chemotherapy for small cell lung cancer. J Inflammation Res. (2025) 18:9343–53. doi: 10.2147/JIR.S517816, PMID: 40692548 PMC12278946

[13] Saal J Bald T Eckstein M Ritter M Brossart P Ellinger J. Early C-reactive protein kinetics predicts immunotherapy response in non-small cell lung cancer in the phase III OAK trial. JNCI Cancer Spectr. (2023) 7. doi: 10.1093/jncics/pkad027, PMID: 37004206 PMC10121335

[14] Sung M Jang WS Kim HR Park JA Lim SM Kim HR. Prognostic value of baseline and early treatment response of neutrophil-lymphocyte ratio, C-reactive protein, and lactate dehydrogenase in non-small cell lung cancer patients undergoing immunotherapy. Transl Lung Cancer Res. (2023) 12:1506–16. doi: 10.21037/tlcr-23-7, PMID: 37577328 PMC10413036

[15] Rudin CM Poirier JT Byers LA Dive C Dowlati A George J. Molecular subtypes of small cell lung cancer: a synthesis of human and mouse model data. Nat Rev Cancer. (2019) 19:289–97. 10.1038/s41568-019-0133-9 PMC6538259 30926931

[16] Nishino M Jackman DM Hatabu H Yeap BY Cioffredi LA Yap JT. New Response Evaluation Criteria in Solid Tumors (RECIST) guidelines for advanced non-small cell lung cancer: comparison with original RECIST and impact on assessment of tumor response to targeted therapy. AJR Am J Roentgenol. (2010) 195:W221–8. doi: 10.2214/AJR.09.3928, PMID: 20729419 PMC3130298

[17] Choi HC Kim JH Kim HS Jung SG Hwang SM Ju SB. Comparison of the RECIST 1.0 and RECIST 1.1 in non-small cell lung cancer treated with cytotoxic chemotherapy. J Cancer. (2015) 6:652–7. doi: 10.7150/jca.11794, PMID: 26078796 PMC4466415

[18] Hutajulu SH Astari YK Ucche M Kertia N Subronto YW Paramita DK. Prognostic significance of C-reactive protein (CRP) and albumin-based biomarker in patients with breast cancer receiving chemotherapy. PeerJ. (2025) 13:e19319. doi: 10.7717/peerj.19319, PMID: 40416620 PMC12103165

[19] Zhang H Dou B Sun X Chen X. Interaction effects between serum 25(OH)D and CRP status on cancer related mortality in adult cancer survivors. Sci Rep. (2025) 15:14798. doi: 10.1038/s41598-025-95931-w, PMID: 40295518 PMC12038049

[20] Gay CM Stewart CA Park EM Diao L Groves SM Heeke S. Patterns of transcription factor programs and immune pathway activation define four major subtypes of SCLC with distinct therapeutic vulnerabilities. Cancer Cell. (2021) 39:346–60 e7. doi: 10.1016/j.ccell.2020.12.014, PMID: 33482121 PMC8143037

[21] Acar C Yuksel HC Sahin G Acar FP Celebi G Gunenc D. C-reactive protein kinetics as prognostic biomarkers in advanced melanoma treated with immune checkpoint inhibitors. Melanoma Res. (2025) 35:232–41. doi: 10.1097/CMR.0000000000001039, PMID: 40202929

[22] Sahin G Acar C Yuksel HC Tunbekici S Acar FP Celebi G. Dynamic blood-based biomarkers predict early response to ipilimumab and nivolumab in advanced melanoma. Clin Transl Oncol. (2025). doi: 10.1007/s12094-025-04024-7, PMID: 40841505

[23] Chiou VL Burotto M. Pseudoprogression and immune-related response in solid tumors. J Clin Oncol. (2015) 33:3541–3. doi: 10.1200/JCO.2015.61.6870, PMID: 26261262 PMC4622096

[24] Yu Y Wang S Su N Pan S Tu B Zhao J. Increased circulating levels of CRP and IL-6 and decreased frequencies of T and B lymphocyte subsets are associated with immune-related adverse events during combination therapy with PD-1 inhibitors for liver cancer. Front Oncol. (2022) 12:906824. doi: 10.3389/fonc.2022.906824, PMID: 35756643 PMC9232255

[25] Iivanainen S Ahvonen J Knuuttila A Tiainen S Koivunen JP. Elevated CRP levels indicate poor progression-free and overall survival on cancer patients treated with PD-1 inhibitors. ESMO Open. (2019) 4:e000531. doi: 10.1136/esmoopen-2019-000531, PMID: 31555483 PMC6735669

