# Supplementary material for: Early C-reactive protein reduction predicts survival in real-world extensive-stage small cell lung cancer treated with first-line adebrelimab-based immunotherapy
Source: Front Oncol. 2025 Nov 5;15:1709336. doi: 10.3389/fonc.2025.1709336 (PMC12626855; doi:10.3389/fonc.2025.1709336)
Supplement: Supplementary file 1 [file DataSheet1.docx]

**Table S1. Baseline characteristics.**

| **Baseline characteristics** | **All patients (n = 35)** |
| --- | --- |
| Age (years), n (%) |  |
| Median (range) | 72 (52-87) |
| ≥65 | 31 (88.6) |
| <65 | 4 (11.4) |
| Gender, n (%) |  |
| Male | 31 (88.6) |
| Female | 4 (11.4) |
| Brain metastasis, n (%) | 11 (31.4) |
| Smoking status, n (%) |  |
| Nonsmoker | 11 (31.4) |
| Former smoker/smoker | 24 (68.6) |
| Number of metastatic organs, n (%) |  |
| ≥2 | 18 (51.4) |
| <2 | 17 (48.6) |
| ECOG PS, n (%) |  |
| 0–1 | 30 (85.7) |
| 2 | 5 (14.3) |
| Adebrelimab, median (IQR) | 4 (3-8) |
| Chemotherapy agents in the cohort, n (%) |  |
| EP | 18 (51.4%) |
| EC | 10 (28.6%) |
| IP | 7 (20.0%) |
| level of systemic inflammation, (mean ± *SD*) |  |
| NLR | 4.97±0.64 |
| PLR | 186.08±20.63 |
| LMR | 2.51±0.23 |
| PAR | 5.70±0.77 |
| SII | 1217.37±425.90 |
| NPR | 0.04±0.01 |
| CAR | 0.95±0.37 |
| CLR | 34.27±11.46 |
| CPR (mg/L) | 30.61±10.30 |
| LDH (U/L) | 329.37±46.29 |

Abbreviations: EP, Etoposide + Cisplatin; EC, Etoposide + Carboplatin; IP, Irinotecan + Cisplatin; NLR, Neutrophil-to-Lymphocyte Ratio; PLR, Platelet-to-Lymphocyte Ratio; LMR, Lymphocyte-to-Monocyte Ratio; PAR, Platelet-to-Albumin Ratio; SII, Systemic Immune-Inflammation Index (Platelets × Neutrophils / Lymphocytes); NPR, Neutrophil-to-Platelet Ratio; CAR, C-reactive Protein-to-Albumin Ratio; CLR, C-reactive Protein-to-Lymphocyte Ratio; CRP, C-reactive Protein; LDH, Lactate Dehydrogenase.

**Table S2. Efficacy of Adebrelimab in** **SCLC patients (n = 35).**

| **Efficacy** | **All patients (n = 35)** |
| --- | --- |
| Complete response (%) | 0 |
| Partial response (%) | 22 (62.8) |
| Stable disease (%) | 5 (14.3) |
| Progressive disease (%) | 8 (22.9) |
| Objective response rate (%, CR, PR) | 22 (62.8) |
| Disease control rate (%, CR, PR, SD) | 27 (77.1) |
| median progression-free survival (months, 95% CI) | 7.10 (5.47, 8.53) |
| median Overall Survival (months, 95% CI) | 15.00 (10.47, 19.53) |


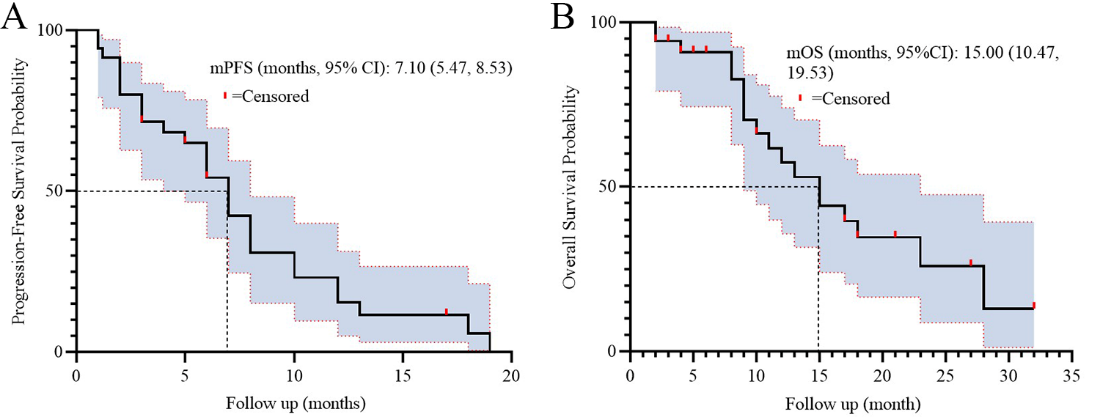


Figure S1. Kaplan-Meier survival curves of PFS (A) and OS (B) in 35 patients.
